# Epidemiologic patterns of injuries treated at the emergency department of a Swedish medical center

**DOI:** 10.1186/s40621-014-0033-0

**Published:** 2015-01-30

**Authors:** Fredrik Röding, Marie Lindkvist, Ulrica Bergström, Jack Lysholm

**Affiliations:** 1 Division of Surgery and Perioperative Sciences, Department of Orthopaedics, Umea University, 90187 Umea, Sweden; 2 Department of Public health and Clinical Medicine, Epidemiology and Global Health, Umea University, 90187 Umea, Sweden; 3 Umea school of business and economics, Department of Statistics, Umea University, Umea, 90187 Sweden; 4 Division of Surgery and Perioperative Sciences, Department of Orthopaedics, Umea University, 90187 Umea, Sweden; 5 Division of Surgery and Perioperative Sciences, Department of Orthopaedics and Department of Public Health and Clinical Medicine, Epidemiology and Global Health, Centre of Quality Registries North Sweden, Umea University, 90187 Umea, Sweden

**Keywords:** Injury, Fracture, Contusion, Strain, Wound, Fall, Epidemiology

## Abstract

**Background:**

The injury spectrum published in the literature has mainly been presented for a certain age group, as elderly or for a certain type of injury, as fracture and often restricted to in-hospital care cases. Our objective was to give an overview of the major types of injuries for all age groups and trends in the adult population.

**Methods:**

We analyzed 68,159 adult injury events, which occurred between 1999 and 2008 and was treated at the Emergency Department of Umea University Hospital. All these injuries are registered in a database. The injuries were analyzed depending on frequency, type of injury, and activity at the time of injury. Incidence rates were calculated using population data from Statistics Sweden.

**Results:**

Injury event incidence varied between 614 (2004) and 669 (2007) per 10,000 persons. The most common injury was a fracture, although contusions and wounds were also frequent. Fractures were responsible for almost three quarters of hospital days related to injury. The risk for fractures increased with age, as did contusions and concussions, whereas sprains decreased with age. Fracture incidence increased among the 50- to 59-year age group for both women and men. Fall-related injuries increased significantly for middle-aged adults. Sports-related and work injuries decreased, while injuries occurring during leisure time increased the most.

**Conclusion:**

A fracture is the most frequent type of injury for adults and accounts for the largest proportion of the trauma care burden. Contusions are also common and responsible for a significant proportion of the in-hospital days. Injuries caused by a fall increased among middle-age adults imply a need for an extension of fall prevention programs.

## Background

Injury is widely known to be a considerable global public health issue. In the EU, more than 250,000 people are killed every year as a result of an injury event and more than 3 million are permanently disabled (Bauer and Steiner 2009). Among males under the age of 65, injury is the most significant cause of reduction of life expectancy and among men 15 to 65 years of age; injury is the leading contributor to health care costs for this age group (Polinder et al. 2012; Swedish Centre for Lessons Learned from Incidents & Accidents 2005). From the 1970s until the end of the 1990s, injury mortality decreased in Sweden, but at the beginning of the new millennium, there was a break in this trend, with an increase in mortality due to injuries (Swedish Centre for Lessons Learned from Incidents & Accidents 2005).

In particular, falls and fractures among the elderly have been the topic of numerous publications. An increase in injury mortality due to falls has been demonstrated among adults over 65 years of age living in the USA (Alamgir et al. 2012; Hu and Baker 2010). During the last few decades of the twentieth century, fractures and severe head injuries among the elderly have increased (Ahmed et al. 2009; Kannus et al. 2007. Kannus et al. 2009; Lönnroos et al. 2006; Pérez et al. 2012; Saveman and Björnstig 2011). However, in the past decade, this trend for hip fractures was reversed in several countries, i.e., Scandinavia, Switzerland, and Ireland (Bergström et al. 2009; Korhonen et al. 2013; Lippuner et al. 2011; McGowan et al. 2012; Stoen et al. 2012).

Unfortunately, the spectrum of injury for adults below the age of 65 has not been thoroughly investigated. With significant changes witnessed in the environment and in human behavior, such as efforts to reduce traffic and occupational safety exposures, it is important to consider the potential implication of these changes on injury trends, especially since exposures to other injury risks seem to have increased with more people participating in physically demanding activities at higher ages (Borodulin et al. 2008; Ng et al. 2011; Petersen et al. 2010). Injury patterns are also likely to change in different stages of life as a result of changes in activities, the environments in which these activities take place and the natural changes in the human body that take place due to ageing and co-morbidities (MacKenzie 2000).

Previous studies that have examined injury patterns have largely focused on specific injuries, such as falls and fractures or specific age groups, like the elderly. However, little is known about the wider spectrum of adult injury patterns. The potential value of this type of overview is that it allows researchers and health professionals to compare trends across age groups and injury categories so that priorities for injury prevention can be developed. The purpose of this study was to compare changes in injury patterns as a whole as well as between age and gender groups using injury data from one particular hospital over a 10-year period.

## Methods

Since 1985, all injury events treated at the Umea University Hospital Emergency Department have been digitally registered in a database. Umea University Hospital is situated in the Northern part of Sweden with varying weather conditions. Summer is present in June, July, and August with temperatures about 20°C. Winter lasts from November until April, when snow and ice is common and the temperature, below 0°. The hospital is responsible for all the emergency care for the city of Umea and its surrounding neighborhoods (145,000 inhabitants and 10,730 km^2^). There is no other hospital in this part of Vasterbotten County. Injuries which have resulted in death prior to arrival at the emergency department are not included.

Upon arrival at the emergency department, an injured person (or a relative) is asked to fill in a questionnaire which provides information about the circumstances of the injury. The questionnaire provides information about the purpose of the digital registry, e.g., research to improve safety and prevent future injuries. By filling it in, they give their informed consent to participate. All questionnaires are collected by the hospital's injury surveillance group.

In cases where information is missing or vague, the information is supplemented and verified with data from medical records, ambulance, and police reports. The database is regularly tested for missing cases by the hospital's patient registry for inpatient cases and by billing information from the emergency department for outpatient cases. During the current period, the proportion of missing cases was found to be 3.6%. From 2006 to 2008, the database also included cases from a unit which purpose was to reduce the patient load at the emergency department (this data accounted for roughly 4% of all injury events during that period).

Permission to use the injury data for research was provided by the registry steering group at the county council and by the Regional Ethics Committee in Umea. The registry is also part of the European Injury Data Base (IDB).

For this study, we examined injury data for all injuries between 1999 and 2008 for adults (20 years of age and older) residing in the A-64 region registered by the Swedish Tax Agency (the inhabitants of Sweden are obliged by law to inform the tax agency about their address of residence within one week after moving to a new area). Visitors from other parts of Sweden or other countries were not included in the analysis.

Since the information gathered in the registry was captured with a high degree of resolution, it was necessary to combine some of the variables into larger injury categories. These were as follows: fracture, contusion, wound, sprain, concussion, and others. Most of the injuries grouped into these injury categories were fairly homogenous. However, in the case of the variable 'wound,' the spectrum of injury was relatively diverse and included simple open wounds engaging the skin and sometimes subcutaneous tissue as well as more severe injuries including muscle, nerve, vascular injuries, and traumatic amputations. Simple open wounds contributed to 86% of the cases classified as a wound.

To classify activities, we used the following categories: work, leisure, road traffic, and sports. The work category is based on the following sub-categories: employment, housework, and education. The road traffic category consists of the sub-categories car and bicycle. To classify the severity of an injury, the injury severity score (ISS) was used. The ISS is an anatomical scoring system that gives an overall score for patients with multiple injuries. The ISS is based on the abbreviated injury scale (AIS). For each body region injured, the AIS gives a number between 1 and 6. The three most injured body regions have their AIS number squared and summed up to give the ISS (Association for the Advancement of Automotive Medicine 2001; Baker et al. 1974).

### Statistics

The incidence per 10-year age groups was calculated by the number of injury events among people in the age group during the specific period divided by the total midyear population in the age group during this period. This was reported as events per 10,000 person-years with a 95% confidence interval. Logistic regression was used to evaluate the trends in risk for injuries as well as activities the individual was engaged in at the time of the injury. The dependant variable was injury (injury = 1; non-injury = 0), and the continuous predictor variable was time (in years). Population data was collected from Statistics Sweden, using the annual midyear population. The data were analyzed using SPSS version 21. In this paper, the word 'significant' is equated to a *p*-value of less than 0.05.

## Results

A total of 68,159 injuries were registered during 1999 to 2008. Thirty-one thousand thirty-three individuals were injured once and 13,889 individuals two or more times. During this time period, the population at risk increased from 102,010 to 109,788. The overall injury incidence varied between 614 (2004) and 669 (2007) emergency department visits per 10,000 inhabitants. There was an annual significant increase in injury incidence of 0.5%. The most common injuries were fractures, which accounted for nearly a quarter of all injury events and almost three quarters of the hospital days attributed to injury treatment (Tables 1 and 2).

**Table 1:** **Injury numbers categorized by injury type and mechanism, for ages 20 and above, during years 1999 to 2008**

**Year**	**1999**	**2000**	**2001**	**2002**	**2003**	**2004**	**2005**	**2006**	**2007**	**2008**
Midyear population	102,010	102,538	103,101	104,005	105,310	106,600	107,919	108,769	109,182	109,788
Incidence per 10,000 persons-years	649	621	634	650	636	614	637	661	669	661
Injuries										
Men	3,711	3,590	3,638	3,769	3,611	3,613	3,776	3,886	3,953	3,899
Women	2,912	2,782	2,901	2,989	3,088	2,929	3,094	3,309	3,350	3,358
Injury category										
Fracture	1,615	1,508	1,701	1,662	1,700	1,561	1,710	1,897	1,896	1,859
Wound	1,557	1,482	1,413	1,489	1,510	1,496	1,554	1,549	1,744	1,729
Contusion	1,142	1,131	1,355	1,497	1,455	1,482	1,509	1,635	1,637	1,745
Sprain	1,111	1,107	1,220	1,292	1,237	1,161	1,213	1,303	1,436	1,345
Concussion	218	271	210	201	200	205	227	247	215	218
Other	980	873	640	617	597	637	657	564	373	361
Injury mechanism										
Fall	2,730	2,700	2,920	3,007	3,011	2,840	3,026	3,299	3,235	3,215
Bicycle	379	360	376	376	336	340	361	384	386	405
Motorvehicle	489	496	524	564	554	496	515	541	607	542
Violence	251	230	217	221	253	232	262	238	284	257
Self harm	78	41	71	52	162	126	112	106	69	57
Injuries from heat, cold, poison, electricity, explosive	136	103	135	100	201	200	153	159	109	121
Overstrain	325	347	333	428	356	398	486	420	449	492
Sport	877	901	882	901	850	857	819	864	846	822
Foreign body	298	270	254	231	189	214	246	191	147	109
Other	1,060	924	827	878	787	839	890	993	1,171	1,237

**Table 2:** **Numbers and proportion of emergency department visits and in-hospital days for major injury types**

**Injury type**	***N*** **(visits)**	**Proportion of ED visits (%)**	***N*** **(days)**	**Proportion of in-hospital days (%)**
Fracture	17,109	25.1	97,958	74.9
Wound	15,523	22.8	11,112	8.5
Contusion	14,588	21.4	5,773	4.4
Sprain	12,425	18.2	4,011	3.1
Concussion	2,212	3.2	2,247	1.7
Other	6,304	9.2	9,661	7.4

### Injuries and age

**Figure 1:**
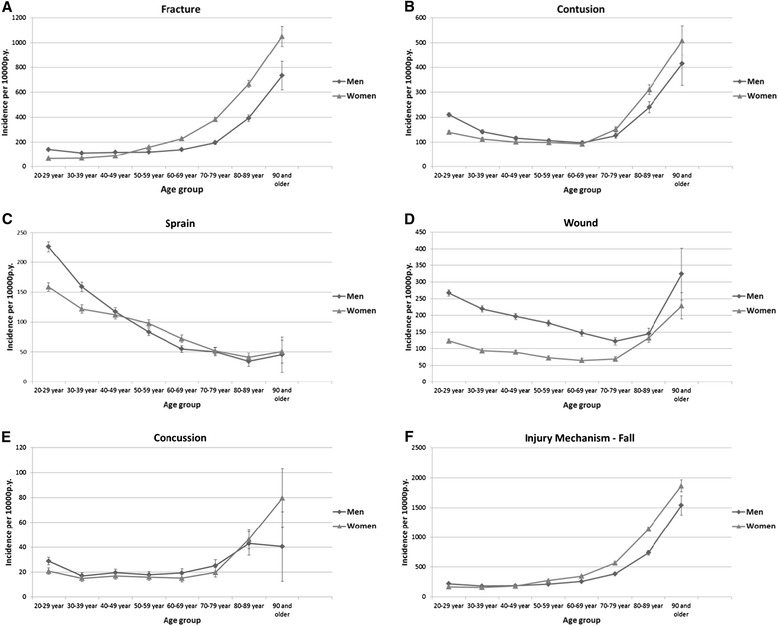
**Incidence for women and men in 10**-**year-age groups (95% confidence interval), for five injury types and falls, 1999 to 2008 (A to F).**

Age was associated with injury incidence for all injury types. An increased incidence of fractures was found with increasing age. The pattern was similar for all other injuries except for sprains, where incidence fell with increasing age. However, wounds were almost as common for young adults, as they were for elderly men (Figure 1). All injuries due to falls increased with increasing age.

### Trends

There was a significant increase in the risk for fractures for both women (4% per year) and men (3% per year) in the ages between 50 and 60. For all other age groups, the risk for fractures was unchanged.

Contusions increased in both women and men in most age groups. There was also a significant increase in the risk of sprains in the age group 40 to 49 in both sexes and in men between the ages of 20 to 29 and 50 to 59. For wounds, trends were more varied and for concussions, a decrease among the oldest age groups was seen. Injuries which were sustained as the result of a fall showed a significant increase in the ages 40 to 70 for women and between 30 and 60 years for men. For the youngest male age group, there was a significant decrease. For individuals over 80 years of age, there was a general increase, but not significant (Tables 3).

**Table 3:** **Trends over time for five major types of injury and fall injuries in different age groups and gender, 1999 to 2008**

**Age group**	**Men**	**Women**	**Men**	**Women**
	Fracture		Contusion	
20 to 29	1.00 (0.98 to 1.02)	1.00 (0.98 to 1.03)	1.02 (1.00 to 1.03)	1.02 (1.00 to 1.04)
30 to 39	0.98 (0.96 to 1.00)	0.99 (0.96 to 1.02)	1.01 (1.00 to 1.03)	1.05 (1.03 to 1.07)
40 to 49	1.02 (1.00 to 1.04)	1.01 (0.99 to 1.04)	1.05 (1.03 to 1.07)	1.05 (1.03 to 1.08)
50 to 59	1.03 (1.01 to 1.05)	1.04 (1.03 to 1.06)	1.05 (1.02 to 1.07)	1.04 (1.01 to 1.06)
60 to 69	1.01 (0.99 to 1.03)	1.01 (1.00 to 1.03)	1.04 (1.01 to 1.07)	1.03 (1.00 to 1.06)
70 to 79	1.00 (0.98 to 1.03)	1.00 (0.98 to 1.01)	1.07 (1.04 to 1.10)	1.04 (1.02 to 1.07)
80 to 89	1.00 (0.97 to 1.02)	1.01 (1.00 to 1.03)	1.05 (1.02 to 1.09)	1.05 (1.02 to 1.07)
90 and older	1.02 (0.96 to 1.08)	1.00 (0.97 to 1.03)	1.08 (1.00 to 1.17)	1.03 (0.99 to 1.08)
Total	1.01 (1.00 to 1.02)	1.02 (1.01 to 1.02)	1.03 (1.02 to 1.04)	1.04 (1.03 to 1.05)
	Wound		Sprain	
20 to 29	0.99 (0.98 to 1.00)	1.00 (0.99 to 1.02)	1.00 (0.98 to 1.01)	1.01 (0.99 to 1.02)
30 to 39	0.99 (0.97 to 1.00)	1.04 (1.01 to 1.06)	1.01 (0.99 to 1.03)	1.01 (0.99 to 1.04)
40 to 49	1.00 (0.98 to 1.02)	1.02 (0.99 to 1.04)	1.04 (1.02 to 1.06)	1.05 (1.03 to 1.07)
50 to 59	1.01 (0.99 to 1.03)	1.03 (1.00 to 1.03)	1.04 (1.01 to 1.06)	1.01 (0.99 to 1.04)
60 to 69	1.04 (1.01 to 1.06)	1.04 (1.01 to 1.08)	1.01 (0.97 to 1.05)	1.01 (0.98 to 1.04)
70 to 79	1.02 (0.99 to 1.05)	1.03 (1.00 to 1.07)	1.03 (0.98 to 1.08)	0.99 (0.95 to 1.04)
80 to 89	1.04 (0.99 to 1.08)	1.02 (0.98 to 1.05)	1.07 (0.98 to 1.17)	1.01 (0.95 to 1.08)
90 and older	1.04 (0.95 to 1.14)	1.05 (0.99 to 1.12)	0.94 (0.74 to 1.18)	1.09 (0.96 to 1.25)
Total	1.00 (0.99 to 1.01)	1.02 (1.01 to 1.03)	1.01 (1.00 to 1.02)	1.02 (1.01 to 1.02)
	Concussion		Mechanism-fall	
20 to 29	0.98 (0.95 to 1.02)	1.04 (0.99 to 1.08)	0.99 (0.97 to 1.00)	1.00 (0.98 to 1.01)
30 to 39	1.02 (0.97 to 1.07)	1.00 (0.94 to 1.06)	0.97 (0.96 to 0.99)	1.00 (0.98 to 1.02)
40 to 49	0.97 (0.92 to 1.02)	1.00 (0.95 to 1.06)	1.02 (1.00 to 1.03)	1.03 (1.01 to 1.05)
50 to 59	0.99 (0.94 to 1.04)	1.04 (0.98 to 1.10)	1.02 (1.00 to 1.03)	1.03 (1.02 to 1.04)
60 to 69	0.98 (0.92 to 1.04)	0.97 (0.91 to 1.04)	1.02 (1.01 to 1.04)	1.03 (1.02 to 1.04)
70 to 79	0.96 (0.89 to 1.02)	1.00 (0.94 to 1.07)	1.02 (1.01 to 1.04)	1.00 (0.99 to 1.02)
80 to 89	0.97 (0.90 to 1.05)	0.89 (0.84 to 0.95)	1.02 (1.00 to 1.04)	1.01 (1.00 to 1.02)
90 and older	0.75 (0.57 to 0.99)	0.90 (0.81 to 1.00)	1.02 (0.98 to 1.07)	1.01 (0.98 to 1.03)
Total	0.98 (0.96 to 1.00)	0.99 (0.97 to 1.01)	1.01 (1.00 to 1.01)	1.01 (1.01 to 1.02)

Odds ratio with 95% confidence interval.

### Activity at the time of the injury

Leisure activities (27%) were the most common source of injury followed by work-related (22%). In the youngest age group (20 to 49 years of age), all types of leisure-associated injuries increased significantly. For wounds, it increased significantly for all age groups. Work-related fractures and sprains decreased in the youngest age group, while work-related contusions increased for this age group. Sports-related injuries remained unchanged overall for all major injury types, aside from wounds which demonstrated a decrease in the 20- to 49-year-old age group (Table 4).

**Table 4:** **Trends over time for four major activity categories preceding the injury**

	**Age group**	**Work**	**Leisure**	**Road traffic**	**Sport**
Fracture	20 to 49 years	0.97 (0.95 to 0.99)	1.02 (1.01 to 1.04)	1.01 (0.99 to 1.03)	0.99 (0.97 to 1.01)
	50 to 69 year	1.01 (0.99 to 1.03)	1.01 (0.99 to 1.03)	1.01 (0.98 to 1.04)	1.02 (0.85 to 1.08)
	70 years and older	0.98 (0.95 to 1.01)	0.99 (0.98 to 1.00)	1.00 (0.95 to 1.04)	0.93 (0.80 to 1.08)
Contusion	20 to 49 years	1.02 (1.00 to 1.04)	1.06 (1.04 to 1.07)	1.02 (1.00 to 1.04)	1.00 (0.99 to 1.02)
	50 to 69 years	0.92 (0.90 to 0.95)	1.02 (1.00 to 1.05)	1.01 (0.98 to 1.05)	0.98 (0.91 to 1.05)
	70 years and older	1.04 (0.99 to 1.10)	1.06 (1.04 to 1.09)	0.99 (0.94 to 1.04)	1.11 (0.94 to 1.32)
Sprain	20 to 49 years	0.98 (0.96 to 1.00)	1.04 (1.02 to 1.06)	1.03 (1.01 to 1.04)	1.00 (0.99 to 1.01)
	50 to 69 years	0.96 (0.94 to 1.00)	0.98 (0.95 to 1.02)	1.04 (1.01 to 1.07)	1.03 (0.97 to 1.09)
	70 years and older	1.00 (0.92 to 1.09)	0.99 (0.95 to 1.04)	1.00 (0.92 to 1.07)	1.18 (0.94 to 1.50)
Wound	20 to 49 years	1.01 (1.00 to 1.02)	1.04 (1.03 to 1.06)	0.95 (0.92 to 0.97)	0.96 (0.94 to 0.98)
	50 to 69 years	1.01 (0.99 to 1.03)	1.06 (1.04 to 1.09)	0.97 (0.93 to 1.02)	1.01 (0.94 to 1.08)
	70 years and older	0.99 (0.95 to 1.03)	1.05 (1.02 to 1.08)	0.99 (0.91 to 1.07)	0.92 (0.75 to 1.14)

Odds ratio with 95% confidence interval. Cases not classified within these four groups are excluded and respond to a total of 26% of the injuries.

### Injury severity

Of the injuries, 69.5% were minor with an ISS of 1 to 3, 24.8% were intermediate with and ISS between 4 and 8, and 5.6% were serious with an ISS of 9 or more. In this analysis, the two first years, 1999 and 2000, were excluded because of a high number of unclassified cases. Over the period from 2001 to 2008, the risk for minor as well as serious injuries increased significantly, with 2% and yearly 4%, respectively, while the intermediate injured decreased with 1%.

## Discussion

### Fractures

One of the most important findings of this study was that fractures were responsible for the biggest burden to the adult trauma care system. This was true for both the number of emergency room visits and for hospital days. Fracture care accounted for three quarters of the total number of hospital days devoted to injury care. This finding should be considered during hospital care planning.

The age-specific incidence showed a steep increase from the age groups 60 to 69 in women and 70 to 79 in men. That has been similarly witnessed in earlier studies (Court-Brown and Caesar 2006). Similar patterns as those identified in our study, for hip fractures (Ahmed et al. 2009; Bergström et al. 2009; Lönnroos et al. 2006) and for distal radius and proximal humeral fractures, have also been highlighted in the literature (Sigurdardottir et al. 2011; Flinkkila et al. 2011)

### Other injuries

Contusions, concussions, and all fall injuries demonstrated similar age and incidence pattern relationships as those found for fractures. Similarly, the risk for injuries resulting in a wound increased in older age groups with a steep increase from the ages of 70 to 79 and 80 to 89 in women and men, respectively. Contusions were responsible for 8.5% of hospital days which indicates that contusions are not a trivial injury and can require extended hospital stays in the elderly.

For injuries other than fractures, there is less information in the literature regarding age-related incidence. A study of the elderly in the USA conducted by Lambers et al. (2012) has shown that contusions to the lower trunk increased with increasing age, starting at around 65 years of age. Kannus showed an increase in fall-induced, severe head injuries in the elderly in Finland between 1970 and 2004 (2007). A similar trend was seen in Italy (Fabbri et al. 2010). In a study from Australia, a significant increase in hospitalizations due to fall-related injuries was found among people aged 65 and older during the years 1999 to 2008 (Watson and Mitchell 2011). This, in combination with the results from our study, indicates that measures for preventing falls in the elderly may not only prevent fractures but also other injuries, which could also reduce health care costs significantly (Sach et al. 2012). The results from our injury analysis also show an increase in injuries due to falls for middle-aged adults, which would imply that fall prevention programs should not only be reserved for elderly individuals but also for younger age groups.

### Time trends

Except for women and men in the 50- to 59-year-old age group who demonstrated an annual 3 to 4% rise, the incidence for a fracture was unchanged. To the best of our knowledge, there is no similar previous study to compare this finding. Previous studies are usually limited to one specific fracture. In comparison to these one specific fracture studies, our findings are consistent with Kannus, who found an unchanged proximal humeral fracture incidence rate in elderly women between 1997 and 2004 (2009). For hip fractures, Korhonen showed a decrease in the incidence after 2000 (2013). Bergström, however, showed that although the age-adjusted incidence of hip fracture declined in adults over 50, both absolute fracture rate and incidence increased in the very old (2009). In a Swiss study, a decrease in the incidence of hospitalization due to hip fracture between 2000 and 2007 was demonstrated; however, an increase in hospitalizations due to other fractures was noted for the same period of time (Lippuner et al. 2011). An explanation to this finding could be that more fractures are treated with surgery which results in more inpatient care. Other studies from Finland demonstrate a similar trend (Huttunen et al. 2012; Mattila et al. 2011).

As well as mirroring incidence trends and relationships that have been similarly noted in the literature, the data reported in our study also points to some novel findings. The increase in fracture incidence for men as well as women in the 50- to 60-year-old age group for the time period covered by this study has never to our knowledge been demonstrated before. Even though an increase in fracture incidence for the same period of time among women at similar ages has been detected in Sweden and Norway, this trend for men is a new finding.

An explanation for the increase in fracture rates for 55- to 65-year-old women between the years 1995 and 2008 has been suggested by Trimpou et al. (2012). They postulated that the decreased use of hormone replacement therapy in women due to negative side effects might be one explanation. In a study from Norway, the incidence for distal forearm fracture among women aged 50 to 64 was reduced by 33% between 1979 and 1999, which lends some support to the theory that hormone replacement therapy may have an effect on fracture incidence (Meyer et al. 2009). The use of hormone replacement therapy in Sweden in women aged 50 to 59 years was 36% in 1999, 27% in 2002, and 9% in 2007 according to the Prescribed Drug Register (Lambe et al. 2010). The protective effect on fracture risk is lost within 5 years from discontinuing the treatment (Yates et al. 2004). However, the fact that men in our study had almost the same rise in incidence over the same time period as women provides some indication that there may be other explanations, as well. Two additional explanations were proposed by Trimpou, either that this group has become more engaged in an active lifestyle or an increased use of tranquilizers, both of which could increase the exposure hazard (2012). As indicated in this study, injuries were most frequently associated with leisure activity. This may support the hypothesis that a more active lifestyle and an increased risk for injuries may go hand in hand (Sundberg 2010).

For contusions, we found an increasing incidence in all age groups, in both men and women between 1999 and 2008. One possible reason could be an increase in propensity for seeking care. Our findings, which demonstrated a yearly increase for the minor injuries, according to ISS, appear to support this conclusion. However, another explanation could relate to a change in behavior, with an increase in adults embracing a physically active lifestyle (Borodulin et al. 2008; Ng et al. 2011; Petersen et al. 2010). In an epidemiological study conducted in Umea, 40-, 50-, and 60-year-old adults were shown to have increased physical activity from 16 to 24% for men and 13 to 30% for women between 1990 and 2007 (Ng et al. 2011). Our analysis of activities preceding an injury showed an increase in injuries associated with leisure activities, while work-related injuries showed a slight decrease. It is possible that this increase in physical activity has increased the risks for injury, as well. Sprains showed trends similar to fractures, with an increase in risk for both women and men in the age groups 40 to 49 and also for men in the age group 50 to 59.

### Strengths and limitations of the study

The strength of this study is that it provides information for a complete population in a well-defined, stable geographic area. Moreover, during this period, no reorganizations within the health care system had been carried out. The database on which this study was based is very circumstantial and has a high degree of validity and coverage, which is not possible for national database registries. There were, however also, some minor limitations. One of the activity variables that correspond to walking showed signs of misclassification and was therefore excluded. In addition, injuries resulting in death prior to admission to the emergency department could not be included in the study. As for all research based on data collected in a database, there are variables, such as lifestyle factors, socioeconomic factors, and comorbidity which would have been valuable to have, but which were not available. Although this can be solved by merging with other databases, this opportunity was not presented within the scope of this study.

## Conclusions

Our results show that fractures are the most frequent injury and are responsible for the greatest burden to the trauma care system. Contusions also contribute to a significant proportion of adult injuries and in-hospital days. Our findings indicate that work injury prevention has likely been successful in lowering rates of workplace injuries. However, the increase in injuries related to leisure time activities, speaks to the need for complementary prevention strategies (Emaus et al. 2011). The data from this study also indicate there is much that can still be done to limit the effects of less severe injuries and to prevent falls. There are many factors which can contribute to injuries and which interact to increase or decrease the risk of injury. The careful examination of contributing factors in injury studies was discussed in 1968 by Haddon (1968). Further studies that carefully dissect the different factors that surround an injury event are needed.
